# Effects of On-Farm Dairy Manure Composting on Tetracycline Content and Nutrient Composition

**DOI:** 10.3390/antibiotics10040443

**Published:** 2021-04-15

**Authors:** Jenna Schueler, Kayla Naas, Jerod Hurst, Diana Aga, Stephanie Lansing

**Affiliations:** 1 Department of Environmental Science and Technology, University of Maryland, College Park, MD 20742, USA; jschueler@cbf.org; 2 Department of Chemistry, University at Buffalo, The State University of New York, Buffalo, NY 14261, USA; kaylanaa@buffalo.edu (K.N.); jerodhur@buffalo.edu (J.H.); dianaaga@buffalo.edu (D.A.)

**Keywords:** antibiotic, antimicrobial, windrow, nitrogen, phosphorus

## Abstract

This study quantified the potential of farm-scale composting to degrade antibiotics in dairy manure. The compost windrow, consisting of sick cow bedding from a 1000-cow US dairy farm, was managed using the dairy farm’s typical practices and monitored for tetracycline and nutrient composition. Samples were collected over 33 days, which was the time from compost pile formation to land application as fertilizer, and analyzed for solids, antibiotics, and nutrient content. Average tetracycline concentrations at the beginning of the study (452 ng/g DW) were lower than at the end of composting (689 ng/g DW), illustrating that antibiotic degradation was not greater than degradation of the compost solids. Total Kjeldahl nitrogen (TKN) increased from 15.3 to 18.4 g/kg during the composting period due to decreases in solids and likely inhibition of N-mineralization due to the presence of antibiotics. The results indicated that antibiotics were not completely degraded when using the farm’s compost pile management techniques, with antibiotics possibly impacting nitrogen transformation in the compost, which should be considered in nutrient management when using sick cow bedding. Additionally, the results showed that antibiotic degradation during farm-scale composting can vary from reported laboratory-scale due to differences in management, composting duration, and temporal conditions, illustrating the need for more extensive on-farm research including common farm practices and real-world conditions.

## 1. Introduction

Composting is a common practice used on farms to manage manure and create nutrient-rich fertilizer. The composting process uses several methods, including static piles, windrows (elongated piles), or in-vessel composting. Piles can be managed with varying levels of intensity, using forced aeration, water addition, and turning within covered or exposed conditions. Additionally, some farms only compost manure solids, while other farms amend compost with other sources of organic matter [1]. Incentives to adopt composting practices include manure volume reduction, odor reduction, weight reduction, and improvement in soil health from compost fertilizer application.

In addition to recycling manure solids, composting has been shown to be an effective treatment for antibiotic mitigation. Studies have shown that composting can decrease tetracycline and its degradation products in manure by up to 70–95% [2,3,4,5]. The concentrations of sulfonamides, such as sulfadiazine, sulfamethazine (SMZ), and sulfamethoxazole, are also reduced or eliminated during composting [6,7]. Kim et al. [8] stated that antibiotic degradation in composting is most likely related to abiotic factors, such as the moisture content, pH, aeration, temperature, carbon-to-nitrogen (C:N) ratio, and nature of the composting substrate.

Previous studies have examined antibiotic degradation during composting with broiler and swine manure substrates; however, there are limited studies examining the fate of antibiotics during the composting of dairy manure, with most prior studies spiking antibiotics into the compost pile, creating artificially high concentrations for the degradation process [7,9,10,11,12,13]. A study by Mitchell et al. [14] examined antibiotics spiked into dairy manure compost and found that concentrations of florfenicol, tylosin, sulfadimethoxine (SDM), and SMZ decreased by more than 95% after 21 days of composting at the pilot-scale, with spiked concentrations ranging from 240 to 3200 ng/g dry weight (DW). Two composting substrates (dairy manure and biosolids) were compared, with greater antibiotic degradation observed using the dairy manure substrate, indicating that substrate type could impact antibiotic degradation in composting; however, tetracyclines (TCs) were not tested [14].

Most literature examining the fate of antibiotics during composting has been conducted in controlled laboratory environments with spiking of high doses of antibiotics; however, these conditions do not reflect those of on-farm composting piles, which fluctuate based on dynamic environmental conditions, pile management practices, and composting duration, with on-farm compost piles generally having lower antibiotic concentrations than spiked laboratory-based studies [2,3,9,15].

Dolliver et al. [10] monitored the degradation of chlortetracycline, monensin, and tylosin during turkey litter composting in a static pile, an intensely managed pile, and in-vessel composting and found no significant differences in degradation between the different composting methods. In contrast, Storteboom et al. [16] compared low-intensity management (LIM) and high-intensity management (HIM) of dairy manure composting and found significantly higher rates of tetracycline dissipation in the HIM compost both at the pilot scale and farm scale. With these varying results and no previous studies investigating composting of un-spiked, sick cow bedding, additional information regarding the fate of antibiotic degradation during farm-scale dairy manure composting under normal farm operating conditions is needed.

Ho et al. [17] correlated the physicochemical properties of the composting process, such as the total nitrogen (TN) and total phosphorus (TP) contents and the C:N ratio, with antibiotic degradation using broiler manure and nine different veterinary antibiotics. It was suggested that the increase in TN in the initial composting phase was due to inhibition of N-mineralization attributable to the presence of antibiotics. Selvam et al. [6] monitored total Kjeldahl nitrogen (TKN) during laboratory-scale composting of swine manure with chlortetracycline, sulfadiazine, and ciprofloxacin and found that antibiotics inhibited nitrogen loss during manure composting. The relationship between nutrients and antibiotic degradation during composting has yet to be explored during dairy manure composting.

The goals of this study were to (1) examine the transformations of tetracycline and its degradation products during the farm-scale composting process of sick cow bedding using existing farm management practices, and (2) monitor nutrient and solid transformations during composting to determine correlations between substrate physicochemical properties and antibiotic degradation. It has been shown that composting characteristics are significantly impacted by management style and intensity, which vary from farm to farm. This study sought to monitor farm-scale antibiotic degradation using sick cow bedding management conditions to better understand how farm management and environmental conditions can impact the efficacy of the composting process. Additionally, the relationship between antibiotic degradation, nutrients, and physical characteristics was further explored. There are few studies that have examined antibiotic fate and nutrient characteristics during dairy manure composting; therefore, this study aimed to fill this knowledge gap and illuminate how the substrate can impact the physicochemical properties of compost and the antibiotic degradation process.

## 2. Results and Discussion

### 2.1. Degradation of Antibiotics during Manure Composting

**Figure 1:**
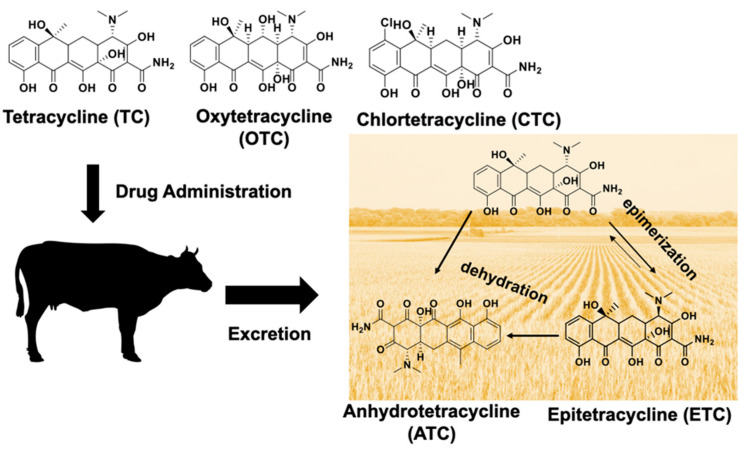
Environmental transformation pathways of tetracycline in the farm-scale context, illustrating the transformation products formed.

TCs can undergo biotic and abiotic transformations that are highly dependent on temperature, pH, and the presence of metals and natural organic matter. For instance, Figure 1 shows the transformation of TC to 4-epitetracycline (ETC) during reversible epimerization and transformation to anhydrotetracycline (ATC) through a dehydration reaction [18]. Analogous transformations can occur for oxytetracycline (OTC) and chlortetracycline (CTC) when excreted in manure. In addition, other transformation products have been reported during bacterial degradation of TCs, including demethylation, dehydrogenation, loss of a carbonyl group, and hydrolysis that leads to the formation of a lactam ring [19].

In this study, during pile formation, TC persisted at concentrations from 13.6 to 690 ng/g DW at the end of the composting monitoring period (Day 33), while SMZ, SDM, and CTC were not detected at any point during the monitoring study (Table 1). TC and ETC were the most abundant antibiotics in all samples, with concentrations ranging from 240 to 710 ng/g DW and 46.4 to 137 ng/g DW, respectively, whereas concentrations of OTC, ATC, and 4-epichlortetracycline (ECTC) did not exceed 67.1 ng/g DW. TC and ECTC were the only analytes that showed significant differences in concentrations between sampling days (*p*-values = 0.0416 and 0.0179, respectively), while concentrations of the transformation products OTC, ETC, and ATC were not significantly different between sampling days (*p*-values = 0.0679, 0.0843, and 0.0706, respectively).

Previous literature has shown high removal of TCs during composting (85–99%) [5]; however, this trend was not reflected in the present study. Initial concentrations of antibiotics in pilot-scale composting in the literature range from 5000 to 22,000 ng/g DW [17,20], which were at least an order of magnitude higher than the initial TC concentrations in the compost pile in the present study (4–450 ng/g DW). Most previous pilot-scale studies spiked large quantities (5000–150,000 ng/g) of antibiotics and observed first-order degradation of antibiotics during the composting process [2,6,9]. The degradation profiles of antibiotics at environmentally relevant concentrations (<1000 ng/g DW) during composting is not well documented. However, Storteboom et al. [16] performed a farm-scale experiment monitoring the degradation of TCs during dairy manure composting for 180 days with weekly turning and consistent watering to maintain a 31 to 36% moisture content. Their compost pile took much longer than the typical 1–4 days to reach peak temperature. The pile took approximately 20 days to reach peak temperature (34 °C) and remained in the mesophilic stage throughout the composting process. The results from Storteboom et al. [16] did show that degradation profiles of TCs followed first-order kinetic degradation, decreasing from 300 ng/g DW to approximately 100 ng/g DW after 30 days, and finally to a nondetectable (ND) concentration after 180 days of composting. The differences between the results in the literature and those in the present study may be due to low starting TC concentrations in the sick cow bedding as well as the relatively short composting period in the present study based on the farm’s compost management practices.

### 2.2. Effect of Temperature on Antibiotic Degradation

**Figure 2:**
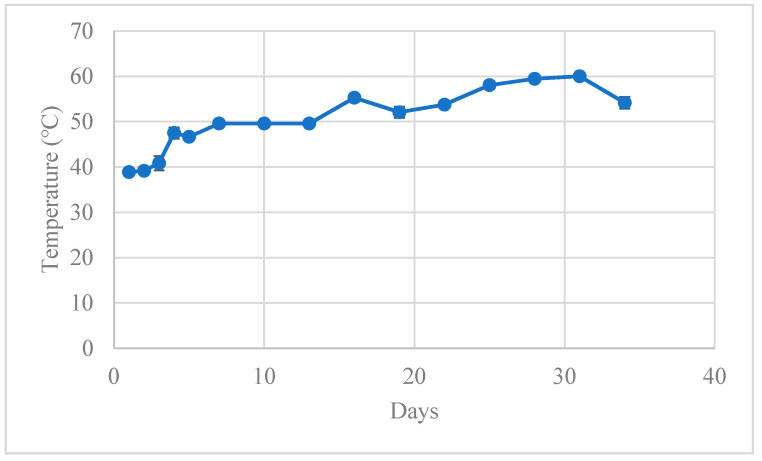
Temperature and standard error from the four temperature probes situated within the compost pile over 33 days, starting from the day of pile creation (Day 0) to the use of the pile on the field as fertilizer (Day 33).

Antibiotic degradation in composting has been linked to microbial activity and absorption, especially for TC [21], yet Youngquist et al. [22] and others [8] stated that abiotic factors are primarily effect antibiotics degradation during the composting process, particularly pile temperature, moisture, and aeration. The TC degradation curves did not follow first-order kinetic degradation during farm-scale composting, despite the temperature reaching a maximum of 60 °C on Day 31 (Figure 2), which was above the 55 °C threshold established by the US Environmental Protection Agency for pathogen destruction [23]. This deviation from first-order degradation was likely due to the relatively gradual temperature increase experienced in the pile and the implementation of frequent turning (every 1–3 days) of the compost pile based on the farm’s normal pile management practices.

In the literature, most compost piles reached peak temperatures (50 to 60 °C) within 1–5 days [4,6,14,24]. Typically, compost pile temperatures begin in the lower end of the mesophilic range (10 to 20 °C) and rise rapidly within the first four days to the thermophilic range (45 to 60 °C). The starting temperature of the compost pile on Day 1 was 38.9 °C, which was already at the high end of the mesophilic temperature range (10 to 40 °C) and approaching the thermophilic range (>40 °C) [25], possibly due to the compost materials consisting of packed bedding for the sick cows, which remains in the barns as bedding for four to six weeks before being removed and placed in windrows. According to US Natural Resources Conservation Services (NRCS), the compost process may start within traditional deep bedded packed materials when in the barn, but it is not a fully composted process [26].

**Figure 3:**
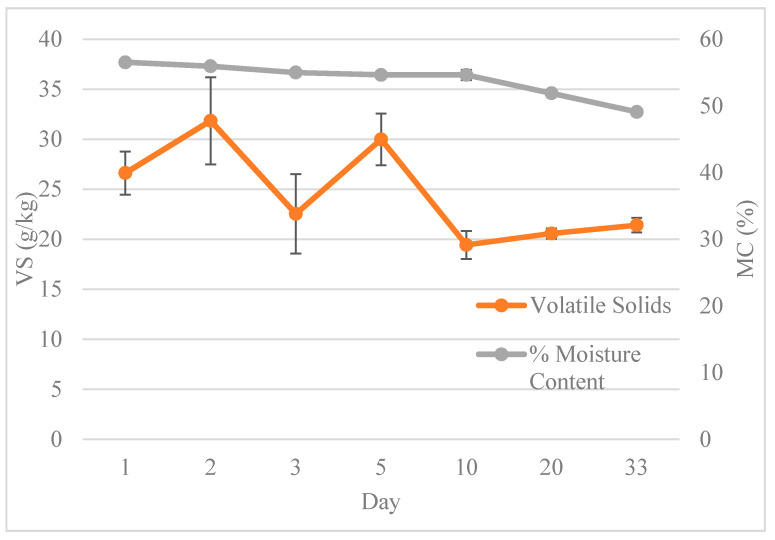
Average volatile solid (VS) concentrations (g/kg) and moisture content (MC) (%) during composting of sick cow bedding from a 1000 cow dairy farm.

The pile in this study was created and monitored during the winter months (end of November through December) when ambient temperatures started off high on Day 1 of pile formation (high temperature of 18.98 °C) but decreased to an average ambient temperature of 4.35 °C during the study period. Cold ambient weather has been shown to decrease the microbial activity during composting, which can limit the temperature rise within the pile [27]. Another factor impacting pile temperature is turning, which ultimately causes the release of heat and moisture from the compost pile [25]. The pile in this present study was intensely managed, with turning every 1–3 days. While the moisture content remained between 50 and 56% (Figure 3) and within the optimal range for compost microbial activity (50 to 70%), turning piles too often prevent sufficient heating of the pile, which may have caused the gradual temperature curve within the pile [1]. Arikan et al. [24] found higher degradation of OTC and ETC with minimally managed piles (no turning) that were covered to increase the temperature to 70 °C compared to uncovered, unturned piles that remained at 36–45 °C. The frequent turning and gradual increase in temperature in the present study (to 60 °C) could indicate inhibition of the microbial community by the TCs present in the compost, as observed in Cessna et al. [28] or inhibition due to the pile management and turning frequency. Both abiotic and biotic factors could have contributed to the low temperature rise and low TC degradation rates observed.

### 2.3. Relationship between Solids and Antibiotic Concentration

The TC, OTC, ETC, and ATC concentrations at the end of the composting period (689, 64.2, 126, and 13.6 ng/g DW, respectively) were higher than at the start of composting (452, ND, 100, and 7.2 ng/g DW, respectively). Composting results in the breakdown of natural organic matter, which can release tightly bound TCs that are strongly adsorbed onto solids with a high organic matter content [29]. In addition, biodegradation can transform TC into OTC, ETC, and ATC (Figure 1).

As shown in Figure 3, the mean moisture content on the last day of the study was 13% lower than Day 1, with a strong linear correlation (R^2^ = 0.9702). The mean volatile solid (VS) value in the pile decreased by approximately 20% from the beginning to the end of the study, but this increase exhibited temporal variation during the study, likely due to turning frequency (R^2^ = 0.3391). The VS concentrations ranged from 20 to 32 g/kg, and similar to the moisture content, showed significant differences over time (*p*-values = 0.0422 for vs. and 0.0076 for moisture content).

### 2.4. Field and Laboratory-Scale Comparisons

Kim et al. [11] monitored TC degradation in an on-farm composting study of swine manure and sawdust over an 80-day period with daily turning and no watering. Temperature was not monitored during this study. These authors compared an 80-day farm-scale composting experiment to a 35-day laboratory-scale experiment, observing that farm-scale TC degradation did not occur as completely as laboratory-scale TC degradation, even with a composting period that was twice as long. The authors concluded that degradation took much longer in farm-scale composting than in laboratory-scale composting.

Extended composting times may be needed at the farm scale to achieve the same levels of degradation seen in laboratory-scale experiments. Interestingly, the decline in TC concentrations at the field-scale did not follow a first-order kinetic degradation pattern both in the present study and in that of Kim et al. [11]. The relatively gradual decline in TC concentration could relate to the frequency of compost turning and the pile temperature. Environmental and management conditions impacting compost piles at the farm scale are more complex than laboratory conditions, which could impact the degradation profile of antibiotics during composting. The duration of farm-scale composting and differences in the pile management intensity could play a key role in the observed degradation profile differences.

### 2.5. Nutrient Transformations during Composting

The TKN concentrations in the compost increased steadily over time, ranging from 15.3 to 18.4 g/kg (Table 2), which corresponded to a decrease in moisture content (Figure 3). The increase in TKN (approximately 20% over the course of the monitoring period) could also be attributed to the presence of TC decreasing N-mineralization due to microbial inhibition. The TP content ranged from 1.2 to 1.8 g/kg (Table 2) and increased by approximately 52% over the course of the monitoring period. Both TKN and TP values showed significant differences between sampling days (*p*-values = 0.0087 and 0.0051, respectively) and showed strong linear correlations with time (R^2^ = 0.98 and 0.93, respectively), which corresponded to decreasing moisture and mass contents of the manure.

The C:N ratios observed in the present study ranged from 13.5 to 12.0 and decreased by approximately 11% over the course of the monitoring period. The C:N ratios of the pile were below the average C:N ratio documented for cow manure and did not change significantly during the study. The ideal starting range of the C:N ratio for composting is 25–35, with a reasonable range extending between 20 and 40. The starting C:N ratio of the pile was lower than the ideal composting range, likely because the manure was composted without carbon amendment to increase the carbon content, as well as the likely occurrence of in-situ composting conditions prior to the compost pile formation.

Cattle manure tends to have relatively low C:N ratio, with an average ratio of approximately 19 [25]. When the C:N ratio is low (<20), carbon is utilized without stabilizing all of the nitrogen, increasing the risk of nitrous oxide emissions to the atmosphere, a gas 300 times more potent than CO_2_ that also has the potential to deplete the ozone layer. Additionally, as compost matures over time, the C:N ratio should decrease due to the loss of CO_2_ from the starting materials exceeding the loss of nitrogen, which could have been the case for the sick cow bedding during its use. If the starting C:N ratio is <15, the rates of carbon and nitrogen loss could be equal, resulting in little change in the C:N ratio over time [25]. In the present study, the C:N ratio was <1:5, but the TKN concentration increased with no significant changes in the C:N ratio (*p*-value = 0.1271).

The 20% increase in TKN over time in the present study could be related to the antibiotics present in the compost, as increases in TKN during manure composting within the presence of antibiotics have been documented in other studies. Selvam et al. [6] examined nutrient trends during a 56-day laboratory-scale composting of sawdust and swine manure that was regularly moistened and aerated and spiked with CTC, sulfadiazine, and ciprofloxacin. The authors observed a 12% increase in TKN during the composting period, from 1.7–1.9%, which was attributed to antibiotic inhibition of N-mineralization. Their C:N ratio declined from 29 to 23 during composting, corresponding to the increase in TKN, which differed from the present study, in which no significant changes in the C:N ratio were observed during the monitoring period.

Additionally, Ho et al. [17] observed an increase in TN (5%) from Day 0 to Day 4 (43 to 45 g/kg) in a 40-day, laboratory-scale broiler manure compost pile that was spiked with doxycycline and several other antibiotics. The moisture content was maintained between 50 and 60%, and the compost was mixed daily during the experiment. This initial increase in TN was followed by a gradual decrease to 38 g/kg (11% lower than the starting concentration) until compost completion. Selvam et al. [6] and Ho et al. [17] implemented pile management practices similar to those in the present study, but both were conducted at the laboratory scale, while the present study was conducted at the field scale and exposed to greater temperature and environmental variability. These environmental factors could explain some of the differences when comparing the laboratory- and field-scale studies presented in this paper, highlighting the need for additional, detailed field-scale studies.

## 3. Materials and Methods

### 3.1. Field Sampling

A compost windrow was created at a 1000-cow dairy farm in the northeastern US. The pile was monitored from November to December 2017 and was managed using the farm’s typical farm management practices. The pile was kept under roof in an open-air pavilion where the farm manages multiple composting windrows and was turned every 2–3 days. The pile was not watered. The compost pile was constructed using recycled packed bedding made from urine and manure solids from the sick and lame cow barns. Sick cows were treated with a 60 L dose of LA200, a commercial antibiotic that contains 200 mg/mL of tetracycline hydrochloride as an active ingredient. Packed bedding remained in barns for 4–6 weeks before removal for composting and was not aerated or turned during this time. Cow manure patties were not removed during the bedding period and were incorporated into the bedding upon removal from the barn for compost pile creation. No additional antibiotics were spiked into the bedding or compost piles. Samples were collected from the pile 1, 2, 3, 5, 10, 20, and 33 days after pile creation.

A 30 to 35 day composting period is typical for this farm’s management throughout the year (regardless of season), with this study’s composting duration of 33 days representing the average period between pile formation and field application for this farm. Previous studies have shown that the majority of microbial degradation occurs in the first 21 days of composting [14]. Four temperature probes were distributed evenly throughout the pile, with two on each side, to monitor the temperature over time. During each sampling event, four randomized grab samples, evenly spaced along the length of the pile, were taken from each side of the windrow (8 samples total) and mixed thoroughly in a sterile bucket to collect a representative composite sample of the pile. This process was repeated three times, resulting in three composite samples for each sampling day. A well-mixed sub-sample of each of the three replicates was used to fill a 50-mL light-sensitive polypropylene Corning centrifuge tube to be used for antibiotic analysis and a plastic sealable bag for solids and nutrient analysis. The composite subsamples were transported back to the laboratory on ice for analysis.

### 3.2. Laboratory Analysis

Samples were analyzed for total solid (TS), VS, TKN, and TP content, and C:N ratio. For all samples, the TS (Method 2540B) and vs. (Method 2540E) concentrations were determined in triplicate using Standard Methods for the Examination of Water and Wastewater within 24–48 h of collection [30]. For TS analysis, triplicate 10.0 mL samples were pipetted into pre-weighed porcelain crucibles and dried at 105 °C until a constant mass was obtained. For vs. analysis, the dried crucibles were placed in a furnace at 550 °C until a constant weight was obtained. The TKN and TP samples were Kjeldahl digested and analyzed on a Lachat autoanalyzer (Quikchem 850; Loveland, CO, USA). TKN was analyzed using QuikChem Method 13-107-06-2-D (rev. 2012), and TP was analyzed using QuikChem Method 13-115-01-1-B (rev. 2006). Total carbon and nitrogen for the C:N ratio were determined using an elemental analyzer (Elementar Vario Max CNS, Elementar Analysensysteme GmbH, Hanau, Germany).

Samples for antibiotic analyses were collected in 50 mL light-sensitive polypropylene Corning centrifuge tubes that were previously washed with 2% 15.9 M nitric acid. The samples were frozen and lyophilized prior to analysis. Samples were extracted and analyzed using solid-liquid extraction by ultrasonication, cleaned and preconcentrated by solid-phase extraction, and quantified using liquid chromatography-tandem mass spectrometry (LC-MS/MS) (Agilent 6410 triple quadrupole, Santa Clara, CA, USA) following the method described in detail in our prior work [31]. Briefly, a 100-mg aliqoute of freeze-dried manure was weighed into a polypropylene centrifuge tube and was spiked with 50 uL of surrogate solution containing demeclocycline (500 ng mL^−1^). Solids were suspended with 5 mL of 20:30:50 acetonitrile–methanol–0.1 M EDTA–McIlvaine buffer (pH = 4; *v*/*v*/*v*), vortexed for 30 s, ultrasonicated for 10 min, and centrifuged at approximately 4000 g for 10 min. The supernatant was decanted into a 500-mL HDPE bottle, and the solids were extracted twice more. Extracts were diluted with 400 mL of NANOpure water, and pH was adjusted to 4.0, followed by solid-phase extraction (SPE) using tandem amino (NH_2_) SPE and hydrophilic-lipophilic balance (HLB) cartridges. The HLB cartridges were eluted with 10 mL of methanol and evaporated to 200 uL under N2 at 30 °C. Extracts were reconstituted to 1 mL with water/methanol (95:5, *v*/*v*) plus 0.1% acetic acid solution. One-point standard addition quantification was performed for each sample. Specifically, each sample was split into two 200 μL portions; the unspiked sample was spiked with the internal standard (10 uL of 500 ng mL^−1^ minocycline) and 10 uL of methanol, and the spiked sample was fortified with the same amount of internal standard mixture and 10 uL of tetracycline mixture containing 50 ng mL^−1^ of each tetracycline analyzed. Both aliquots were centrifuged at 7000× *g* for 5 min to remove any fine particles from the final extract before analysis by LC-MS/MS using positive-mode electrospray ionization. The following antibiotic compounds were analyzed: TC, ETC, OTC, ATC, CTC, and ECTC. Concentrations of antibiotics are presented in ng/g DW of manure. The detection limit of TC was 2.7 ng/g DW, with the detection limit of the transformation products ranging from 1.3 ng/g DW (ATC) to 13 ng/g DW (CTC). Concentrations below the detection limit were considered non-detectable (ND).

### 3.3. Statistical Analysis

A Kruskal–Wallis nonparametric ANOVA test was used to determine significance differences in antibiotics, nutrients, and solids, and in the moisture content between the different sampling events. All analyses were conducted using an alpha level of 0.05. All statistical tests were performed in Microsoft Excel using the Analysis Toolpak and the StatFi Excel addition.

## 4. Conclusions

Antibiotic degradation results observed during composting at the laboratory and pilot scales may not be indicative of behavior at the farm scale as composting times in the field may be shorter and less predictable. The presence of antibiotics in a compost substrate could also impact nutrient trends through inhibition of N-mineralization, which could affect farm nutrient management plans. In this study, total TC concentrations did not decrease but increased as moisture levels decreased, indicating that solid degradation was occurring at a faster rate than antibiotic degradation. A first-order reaction degradation rate was not observed due to the complexity of on-farm composting. The compost pile consisted of sick cow bedding without spiked antibiotics and organic matter, and antibiotic degradation likely occurred during bedding use before compost pile formation. Further research is needed at the field-scale to better understand the impact of management and environmental conditions on antibiotic degradation and nutrient management, which do not directly correlate with results from laboratory-scale and antibiotic spiking studies in the literature.

## Figures and Tables

**Table 1:** Average antibiotics in ng/g dry weight (DW) for a sick cow bedding compost windrow over a 33-day period. The antibiotics analyzed include oxytetracycline (OTC), 4-epitetracycline (ETC), anhydrotetracycline (ATC), 4-epichlortetracycline (ECTC), and tetracycline (TC).

Day	OTC (ng/g DW)	ETC (ng/g DW)	ATC (ng/g DW)	ECTC (ng/g DW)	TC (ng/g DW)
1	ND *	100 ± 13	7.2 ± 1.1	4.3 ± 0.5	452 ± 71
2	ND *	125 ± 6	11.0 ± 0.6	2.1 ± 0.6	569 ± 5
3	ND *	73 ± 27	7.5 ± 2.8	1.9 ± 0.5	374 ± 136
5	32.1 ± 8.7	72 ± 3	5.4 ± 0.3	ND *	341 ± 27
10	27.6 ± 5.2	125 ± 29	10.4 ± 2.2	ND *	638 ± 72
20	67.1 ± 5.8	137 ± 6.1	13.1 ± 1.2	ND *	684 ± 53
33	64.2 ± 4.2	126 ± 8.1	13.6 ± 1.4	ND *	689 ± 58

* ND indicates a nondetectable concentration.

**Table 2:** Average and standard error of the total Kjeldahl nitrogen (TKN) and total phosphorus (TP) contents and the carbon-to-nitrogen (C:N) ratio of the compost over time.

Day	TKN (g/kg)	TP (g/kg)	C:N
1	15.3 ± 0.2	1.15 ± 0.07	13.5 ± 0.2
2	15.6 ± 0.2	1.30 ± 0.01	12.9 ± 0.1
3	15.8 ± 0.2	1.30 ± 0.03	12.9 ± 0.1
5	16.1 ± 0.1	1.36 ± 0.01	12.0 ± 0.7
10	16.5 ± 0.4	1.43 ± 0.02	12.7 ± 0.3
20	17.4 ± 0.4	1.56 ± 0.04	12.9 ± 0.0
33	18.4 ± 0.7	1.75 ± 0.11	12.0 ± 0.1

## Data Availability

The data presented in this study are available within the article.
